# Assessment of Autoregulation of the Cerebral Circulation during Acute Lung Injury in a Neonatal Porcine Model

**DOI:** 10.3390/children11050611

**Published:** 2024-05-20

**Authors:** Asli Memisoglu, Martha Hinton, Yasser Elsayed, Ruth Graham, Shyamala Dakshinamurti

**Affiliations:** 1 Biology of Breathing Theme, Children’s Hospital Research Institute of Manitoba, Winnipeg, MB R3E 3P4, Canada; acinarmemisoglu@gmail.com (A.M.); martha.hinton@umanitoba.ca (M.H.); 2 Department of Physiology, University of Manitoba, 745 Bannatyne Ave., Winnipeg, MB R3E 0J9, Canada; 3 Section of Neonatology, Department of Pediatrics, Women’s Hospital, Health Sciences Centre, 665 William Ave., Winnipeg, MB R3E 0L8, Canada; yelsayed@hsc.mb.ca; 4 Departments of Anesthesiology, Perioperative and Pain Medicine, Health Sciences Centre, 671 William Ave., Winnipeg, MB R3E 0Z3, Canada; rgraham@hsc.mb.ca

**Keywords:** acute lung injury, cerebral autoregulation, near-infrared spectroscopy, neonate, piglet

## Abstract

In neonates with acute lung injury (ALI), targeting lower oxygenation saturations is suggested to limit oxygen toxicity while maintaining vital organ function. Although thresholds for cerebral autoregulation are studied for the management of premature infants, the impact of hypoxia on hemodynamics, tissue oxygen consumption and extraction is not well understood in term infants with ALI. We examined hemodynamics, cerebral autoregulation and fractional oxygen extraction, as measured by near-infrared spectroscopy (NIRS) and blood gases, in a neonatal porcine oleic acid injury model of moderate ALI. We hypothesized that in ALI animals, cerebral oxygen extraction would be increased to a greater degree than kidney or gut oxygen extraction as indicative of the brain’s adaptive efforts to increase cerebral oxygen extraction at the expense of splanchnic end organs. Fifteen anesthetized, ventilated 5-day-old neonatal piglets were divided into moderate lung injury by treatment with oleic acid or control (sham injection). The degree of lung injury was quantified at baseline and after establishment of ALI by blood gases, ventilation parameters and calculated oxygenation deficit, hemodynamic indices by echocardiography and lung injury score by ultrasound. PaCO_2_ was maintained constant during ventilation. Cerebral, renal and gut oxygenation was determined by NIRS during stepwise decreases in inspired oxygen from 50% to 21%, correlated with PaO_2_ and PvO_2_; changes in fractional oxygen extraction (ΔFOE) were calculated from NIRS and from regional blood gas samples. The proportion of cerebral autoregulation impairment attributable to blood pressure, and to hypoxemia, was calculated from autoregulation nomograms. ALI manifested as hypoxemia with increasing intrapulmonary shunt fraction, decreased lung compliance and increased resistance, and marked increase in lung ultrasound score. Brain, gut and renal NIRS, obtained from probes placed over the anterior skull, central abdomen and flank, respectively, correlated with concurrent SVC (brain) or IVC (gut, renal) PvO_2_ and SvO_2_. Cerebral autoregulation was impaired after ALI as a function of blood pressure at all FiO_2_ steps, but predominantly by hypoxemia at FiO_2_ < 40%. Cerebral ΔFOE was higher in ALI animals at all FiO_2_ steps. We conclude that in an animal model of neonatal ALI, cerebrovascular blood flow regulation is primarily dependent on oxygenation. There is not a defined oxygenation threshold below which cerebral autoregulation is impaired in ALI. Cerebral oxygen extraction is enhanced in ALI, reflecting compensation for exhausted cerebral autoregulation due to the degree of hypoxemia and/or hypotension, thereby protecting against tissue hypoxia.

## 1. Introduction

Neonatal acute lung injury (ALI) is characterized by extensive lung inflammation and surfactant catabolism, leading to lung dysfunction and edema, which can progress to hypoxemic respiratory failure and acute respiratory distress syndrome (ARDS). Neonatal ARDS was redefined by the Montreux consensus in 2017 as “extensive lung inflammation of acute onset in infants from birth to 44 weeks postmenstrual age, triggered by known or suspected clinical insults other than respiratory distress syndrome (RDS), transient tachypnoea of the neonate (TTN), genetic surfactant deficiencies, congenital lung anomalies, pulmonary edema caused by congenital heart diseases, atelectasis or local effusions” [1]. Lung injury may be direct or by indirect extrapulmonary processes such as sepsis, asphyxia or inflammation. Clinical manifestations include decreased lung compliance, heterogeneous atelectasis, intrapulmonary right-to-left shunt, ventilation perfusion mismatch and hypoxemia [1]. Diagnostic criteria include the bilateral presence of diffuse, irregular pulmonary opacities or infiltrates, as well as non-cardiogenic pulmonary edema and hypoxemia [1]. During the COVID pandemic, rare severe cases of neonatal ARDS were observed, prompting greater awareness of alternate pathways to severe respiratory failure in newborns with an impetus to develop management strategies to reduce its severity and ensure optimal patient outcomes [2]. 

The main approach to the treatment of neonatal hypoxic respiratory failure resulting from ALI is to provide adequate oxygen with appropriate ventilator support. However, numerous studies have shown that oxygen use and oxidative stress are closely related in neonates [3,4,5], and both term and preterm infants are at risk from oxidative injury-induced disease caused by inappropriate O_2_. For this reason, it is suggested to target lower oxygen levels in neonates with ALI to prevent oxygen toxicity while preserving vital organ function. While the American Academy of Pediatrics and European guidelines agree on a 90–94% SaO_2_ target for preterm infants born before 32 weeks of gestational age [4,6], there is a lack of clinical trials to recommend target peripheral arterial saturation (SaO_2_) ranges for term infants with hypoxemic respiratory failure from ALI. Moreover, the effects of targeted SaO_2_ ranges on hemodynamics, oxygen consumption and extraction, and overall organ performance have not been clearly delineated. The optimal range of PaO_2_ (or SaO_2_) should ensure adequate oxygen delivery to vital organs to avoid anerobic metabolism and minimize pulmonary vascular resistance but also limit oxidative stress. 

Reliance on peripheral SaO_2_ monitors alone to ensure adequate tissue-level oxygenation can be challenging due to the respiratory instability of sick neonates. An approach that integrates SaO_2_ monitoring and an assessment of hemodynamics using targeted neonatal echocardiography (TNE) with cerebral and regional tissue oxygenation using near-infrared spectroscopy (NIRS) may be more helpful to delineate a safe range of PaO_2_ and oxygen saturation in this patient population. 

TNE provides a longitudinal assessment of myocardial performance, myocardial filling and systemic and pulmonary blood flow and can provide an estimate of the degree of pulmonary hypertension, if present [7]. Near-infrared spectroscopy (NIRS) permits continuous, non-invasive measurement of the oxygenation state of the underlying parenchyma, weighted to the venous contribution, and can be placed to interrogate brain, kidney, mesentery, or limb musculature [8]. The cerebral saturation (rcSO_2_) obtained reflects the balance between oxygen delivery (determined by cardiac output, perfusion pressure, oxygen content and hemoglobin (Hb)) and demand (determined by the cerebral metabolic rate) but is also modulated by CO_2_-induced changes in vascular reactivity, temperature and anesthetic/sedative effects. As such, in the clinical setting, a change in rcSO_2_ value can only be interpreted within the context of all confounding influences.

Given the complexity of the potential interactions, preliminary exploration in an animal model should provide the baseline range of arterial saturations that maintain adequate cardiac function and oxygen delivery to vital organs when perfusion pressures, CO_2_, temperature and metabolic demand can be determined concurrently. To address the research question: At what oxygen saturation threshold does cerebral oxygen delivery become impaired in neonatal ALI, we have developed a neonatal porcine oleic acid (OA) lung injury model that reliably produces a moderate, but stable, level of lung injury [9]. Using this model, we determined simultaneous cerebral, renal and intestinal oxygen saturations using multiple NIRS sensors combined with cardiac function assessed by transthoracic TNE during controlled ventilation and stepwise decreases in inspired oxygen from 50% to 21% (SaO_2_ > 95 to <80%). We hypothesized that below a defined PaO_2_/systemic oxygen saturation threshold, cerebral oxygen extraction would be increased to a greater degree than that of the renal or intestinal beds, indicative of the brain’s adaptive efforts to increase cerebral oxygen extraction at the expense of mesenteric end organs. Secondarily, we sought to determine the saturation threshold below which oxygen delivery would be unable to meet tissue aerobic demand.

## 2. Materials and Methods

The experimental protocol (#21-020, AC 11681) was approved by the University of Manitoba Animal Ethics Committee per Canadian Council on Animal Care guidelines. Fifteen piglets (*Sus scrofa familiaris*) were obtained from a pathogen-free farm supplier at 5 days age and randomized to either control (N = 8) or oleic acid lung injury groups (N = 7). Animals were sedated with intramuscular ketamine/xylazine and atropine (10/1/0.1 mg/kg), followed by inhalational induction with 8% sevoflurane before intubation with a 3.0 cuffed endotracheal tube. After placement of a peripheral intravenous catheter, anesthesia was switched to continuous total intravenous anesthesia (TIVA) with propofol/ketamine (200–250/2.5–5 mg/kg/h) to minimize changes in cerebrovascular reactivity and cardiovascular depression associated with inhalational anesthesia. Anesthesia was titrated by lack of response to surgical stimulation. To prevent spontaneous respiratory efforts during respiratory mechanic measurements, paralysis with rocuronium 1 mg/kg was administered immediately prior to the initial measurement period. A bolus of Lactated Ringers (10 mL/kg) was infused over 20 min, followed by maintenance fluids (D10W at 4 mL/kg/h) for the duration of the study. Continuous mechanical ventilation was initiated in assist control volume guarantee mode with tidal volume (TV) 6 mL/kg, positive end-expiratory pressure (PEEP) 6 cm H_2_O, inspired oxygen (FiO_2_) 50%, inspiratory time (Ti) 0.3 and the respiratory rate (RR) adjusted to maintain arterial partial pressures of carbon dioxide (PaCO_2_) within a normal range. Airway pressure, flow and TV were measured by neonatal pneumotach. Femoral arterial, inferior vena cava (IVC) and superior vena cava (SVC) cannulae were placed via cutdowns and the wounds were infiltrated with bupivacaine 0.25% for analgesia. A 24 g intravenous catheter was placed in the SVC without ligature to minimize interference to cerebral venous return. Electrocardiograph, arterial blood pressure, central venous pressure, peripheral O_2_ saturation and core temperature were continuously monitored; normothermia was maintained by a warm-air heated blanket.

After shaving the skin at each site, neonatal NIRS sensors (FORE-SIGHT^®^, Casmed, Irvine, CA, USA) were placed over the anterior forehead and lower right abdominal wall to monitor cerebral and mesenteric saturations, respectively, while ultrasound siting was performed to ensure correct placement of a renal NIRS probe posteriorly. To minimize ambient light interference with NIRS readings, the sensors were covered with gauze and secured to the skin with tape.

Animals were allowed to stabilize for 15 min after line placement. Baseline assessment was then performed, which included cerebral, gut and renal NIRS measurements, arterial and venous blood gas analysis, and respiratory system mechanics determined from continuously recorded airway pressure and volume loops. Baseline cardiovascular hemodynamics included heart rate (HR), mean arterial (mBP) and central venous blood pressure (CVP) and a hemodynamic echocardiography assessment was obtained from apical four-chamber, subcostal and parasternal views of the heart using a GE Vivid e9 machine with 12 MHz high-frequency probe. Pulmonary aeration was determined by lung ultrasound imaging (POCUS) in the supine position using three anterior and one posterior regions for each lung.

In animals randomized to receive lung injury, 0.2 mL OA, emulsified in 2 mL normal saline, was infused via jugular venous catheter over 20 min as previously described [9,10]. Epinephrine up to 10 μg/kg/min was titrated to support hemodynamics during OA administration. Control animals received a sham infusion of 2 mL normal saline alone. We have previously demonstrated that this protocol can reliably produce a stable, but moderate, level of lung injury [9]. After administration, the animals were maintained for 1 h to permit evolution of their lung injury to a stable plateau, determined by blood gases and respiratory mechanics; at 1 h post injury, all baseline measurements were repeated.

A stepwise decrease in inspired oxygen (40%, 30%, 21%) was then performed with serial blood gases and NIRS determinations at each step. At the end of the experiment, the animals were euthanized with central injection of concentrated KCL 100 mEq/kg.

### 2.1. Data Analysis

Respiratory system mechanics were determined from continuously recorded airway pressure and volume loops. Pulmonary ventilation perfusion (V:Q) matching and intrapulmonary shunt fraction were calculated using software integrating established relationships between stepwise variations in FiO_2_ and measured SaO_2_, predicted for different levels of pulmonary shunt fraction (Qs/Qt) in human neonates. Echocardiogram and US images were analyzed post hoc by an investigator blinded to treatment group assignment (AM) and scored following standardized criteria [7]. We utilized a modified lung ultrasound scoring index described previously [11]. Anterior lungs were divided into upper, middle and lower zones; the posterior lung was assessed in a single posterolateral zone. In each zone, an ultrasound score of 0 = only horizontal A-lines; 1 = ≥3 well-spaced B-lines; 2 = crowded B-lines and/or subpleural consolidation; 3 = extended consolidation, giving a maximum ultrasound score of 24 for each animal.

Cerebral, gut and renal NIRS values were correlated with arterial PaO_2_, regional venous PvO_2_ and mBP. Regional fractional oxygen extraction (FOE) was calculated from arterial and NIRS values according to the formula FOE = (SaO_2_ − rSO_2_)/SaO_2_.

The proportion of cerebral autoregulation impairment attributable to blood pressure, and to hypoxemia, was calculated by established methods [12,13] using a computational algorithm integrating existing published nomograms for cerebral flow regulation with mBP, PaO_2_ and PaCO_2_ [14,15,16] to detect the degree of variation from these nomograms in experimental data.

### 2.2. Statistical Analysis

All data are presented as mean ± SE; animal number (N) or independently measured data points within experiments (n) were compared where relevant. Data were analyzed by one-way ANOVA for repeated measures with Tukey’s post hoc test for comparisons between groups and time points, with a *p* < 0.05 considered significant. Correlations between mBP, PaO_2_ and PvO_2_ and regional NIRS were analyzed by nonlinear regression.

## 3. Results

Fifteen female animals were initially randomized to control (sham infusions; N = 8) or lung injury groups (N = 7). Two animals in the control group showed evidence of respiratory compromise at baseline with hypoxemia and hemodynamic instability and were removed from the study, leaving six control and seven OA-injured animals (average weight 2.1 ± 0.1 kg) for further analysis.

### 3.1. Hemodynamics

Average heart rate, mean blood pressure, central venous pressure and temperature were not affected by OA-induced lung injury and remained stable throughout the study period (Table 1). The decrease in perfusion index observed after OA injury did not reach statistical significance.

### 3.2. Indices of Lung Injury

**Figure 1:**
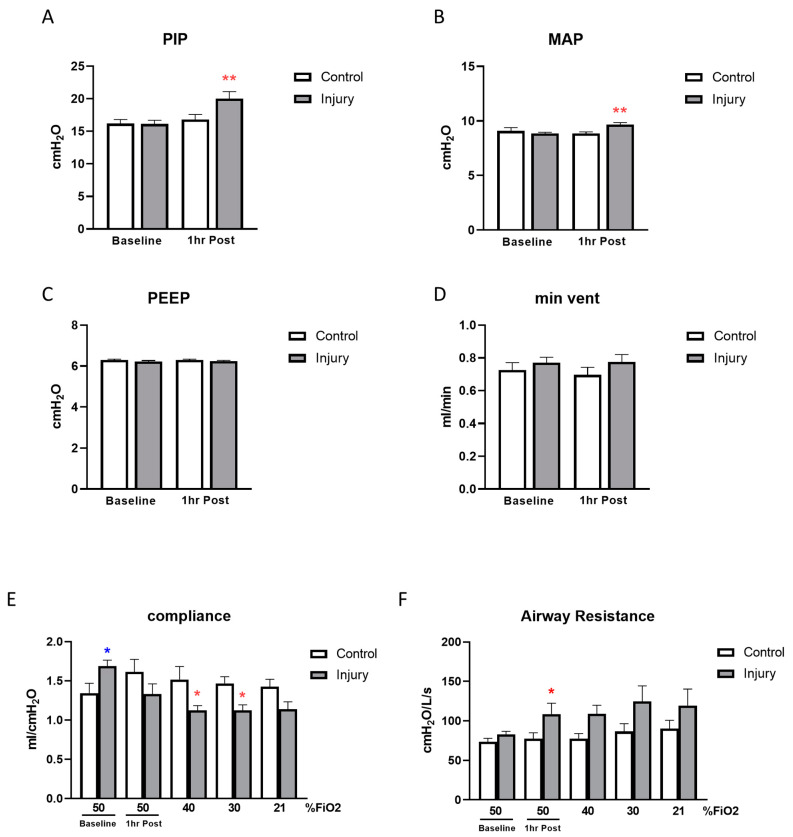
Ventilation parameters and lung mechanics. Peak inspiratory pressure (PIP; (**A**)), mean airway pressure (MAP; (**B**)), positive end expiratory pressure (PEEP; (**C**)) and minute ventilation were compared at baseline and 1 h post lung injury or sham (**D**). Compliance (**E**) and airway resistance (**F**) were measured at baseline, 1 h post treatment and during FiO_2_ stepdown from 50 to 40, 30 and 21%. (N = 6–7, * *p* < 0.05, ** *p* < 0.01).

**Figure 2:**
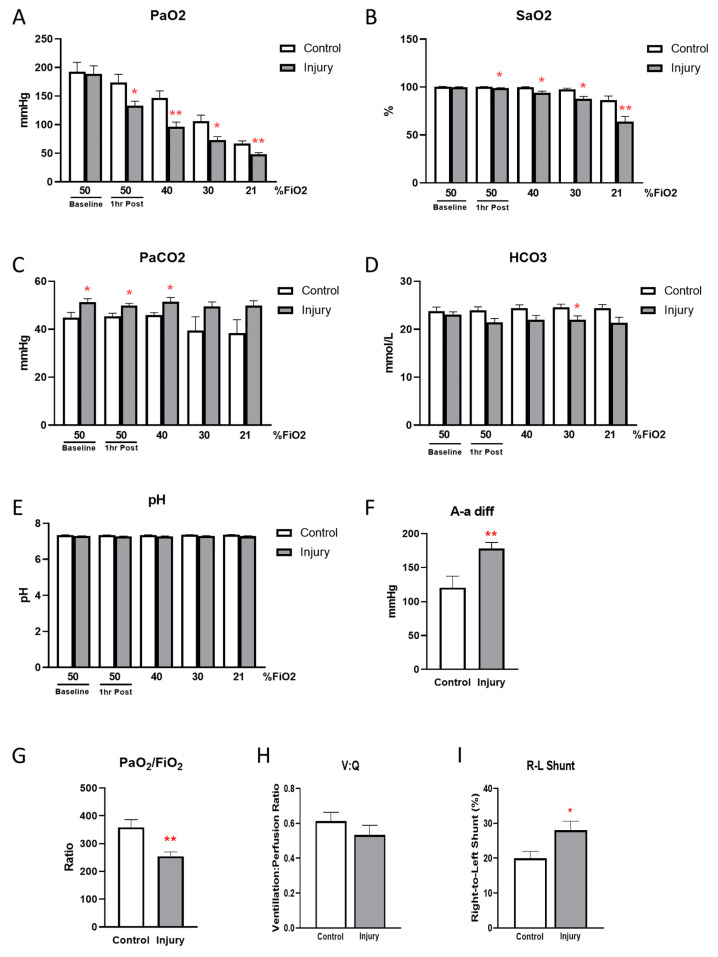
Confirmation of lung injury by blood gases. PaO_2_ (**A**), SaO_2_ (**B**), PaCO_2_ (**C**), bicarbonate (HCO_3_; (**D**)) and pH (**E**) were measured at baseline, 1 h post treatment and during FiO_2_ stepdown from 50 to 40, 30 and 21%. Alveolar–arterial difference (A-a diff; (**F**)), PaO_2_:FiO_2_ ratio (**G**), ventilation to perfusion ratio (V:Q) (**H**) and right to left (R-L) shunt fraction (**I**) were calculated and compared. (N = 6–7, * *p* < 0.05, ** *p* < 0.01).

**Figure 3:**
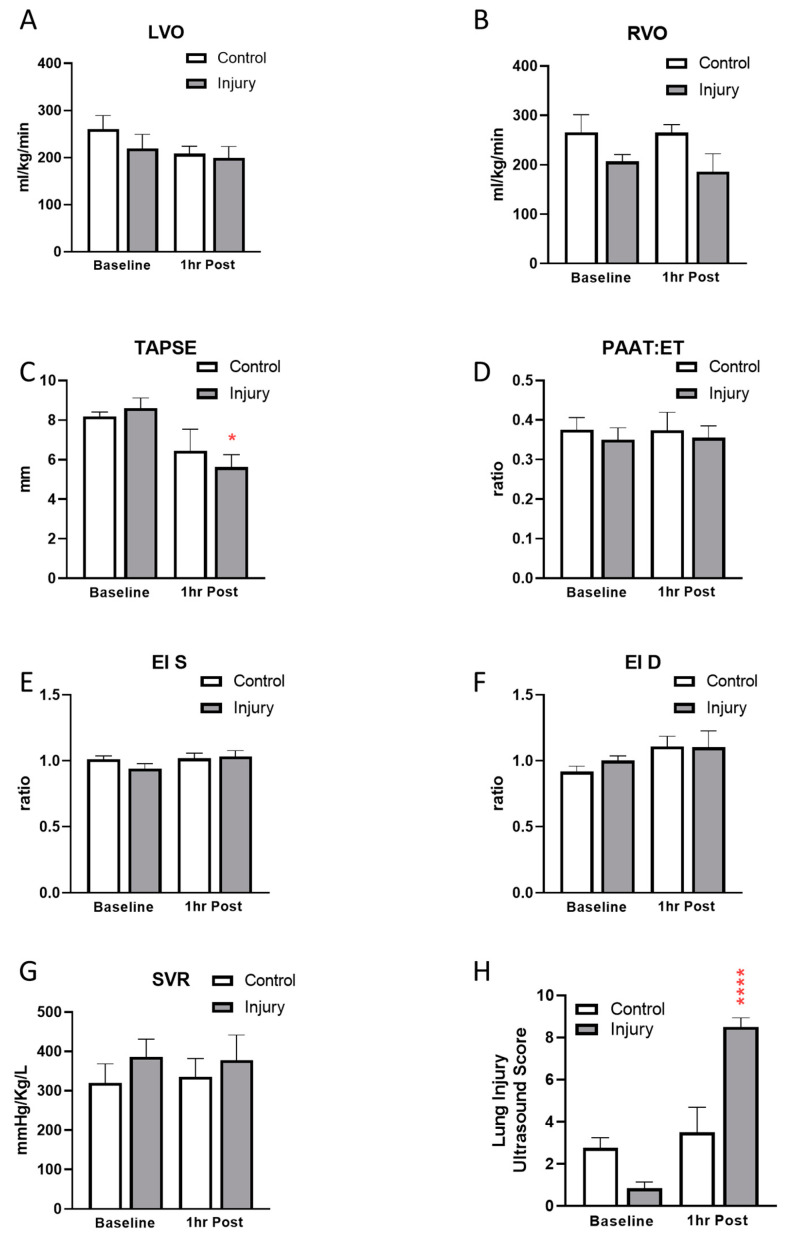
Echocardiography and lung ultrasound. Left (LVO; (**A**)) and right (RVO; (**B**)) ventricular outputs, tricuspid annular plane systolic excursion (TAPSE; (**C**)), pulmonary artery acceleration ratio to ejection time (PAAT:ET; (**D**)), systolic (**E**) and diastolic (**F**) eccentricity indices (EI) and systemic vascular resistance (SVR; (**G**)) were measured at baseline and 1 h post lung injury or sham. Lung injury (**H**) was measured by ultrasound and a composite score from both lungs was compared at baseline and 1 h post lung injury or sham. (N = 6–7, * *p* < 0.05, **** *p* < 0.0001).

Examination of the 1 h post injury respiratory mechanics, blood gases and lung ultrasound, seen in Figure 1, Figure 2 and Figure 3F, confirms the establishment of a moderate degree of acute lung in the OA-injured group of piglets.

Figure 1 provides a comparison of ventilation parameters and lung mechanics. With VT fixed at 6 mL/kg in both groups, PIP, MAP, PEEP and minute ventilation (MV) were unchanged in control animals throughout the study period (Figure 1). Both PIP and MAP were significantly elevated post OA injury (Figure 1A,B) with no change in PEEP and minute ventilation (Figure 1C,D). In the OA-injured group, static lung compliance decreased (Figure 1E) and airway resistance increased (Figure 1F) post injury compared to control despite significantly elevated compliance at baseline.

Arterial blood gas analysis revealed a significant decrease in PaO_2_ with OA injury (Figure 2A). PaO_2_ fell in both groups during O_2_ step-down from 50 to 21%, but more significantly so in the OA-injured group. Arterial oxygen saturation decreased significantly with OA injury, while arterial saturation was unchanged from baseline in control animals until FiO_2_ decreased to 21% (Figure 2B). Arterial PCO_2_ (Figure 2C) was elevated and bicarbonate (Figure 2D), which remained stable during FiO_2_ step-down, tended to be lower, even at baseline, for the OA-injured group. Arterial pH was unchanged by injury or step decrease in FiO_2_ in either group (Figure 2E).

Oxygenation defects were identified in the OA-injured group by a significant increase in the alveolar–arterial difference (A-a diff; Figure 2F), a significantly decreased P/F ratio (PaO_2_:FiO_2_; Figure 2G), a slight drop in the ventilation–perfusion ratio (V:Q; Figure 2H) and a marked increase in intrapulmonary shunt fraction (R-L shunt; Figure 2I).

Lung ultrasound scores and cardiac TNE values are shown in Figure 3. Lung ultrasound scores were low in the control group and unchanged at 1 h, while scores increased significantly 1 h post OA injury (Figure 3F). With respect to cardiac indices, we found no significant change in either left or right ventricular output (Figure 3A,B), but there was a significant decrease in tricuspid annular plane systolic excursion following OA injury (TAPSE; Figure 3C). Normalized pulmonary arterial acceleration time (PAAT:ET; Figure 3D), systolic and diastolic eccentricity indices (Figure 3E,F, respectively) were unaltered, and systemic vascular resistance (SVR; Figure 3G) remained unchanged from baseline in both groups.

### 3.3. NIRS Assessment

**Figure 4:**
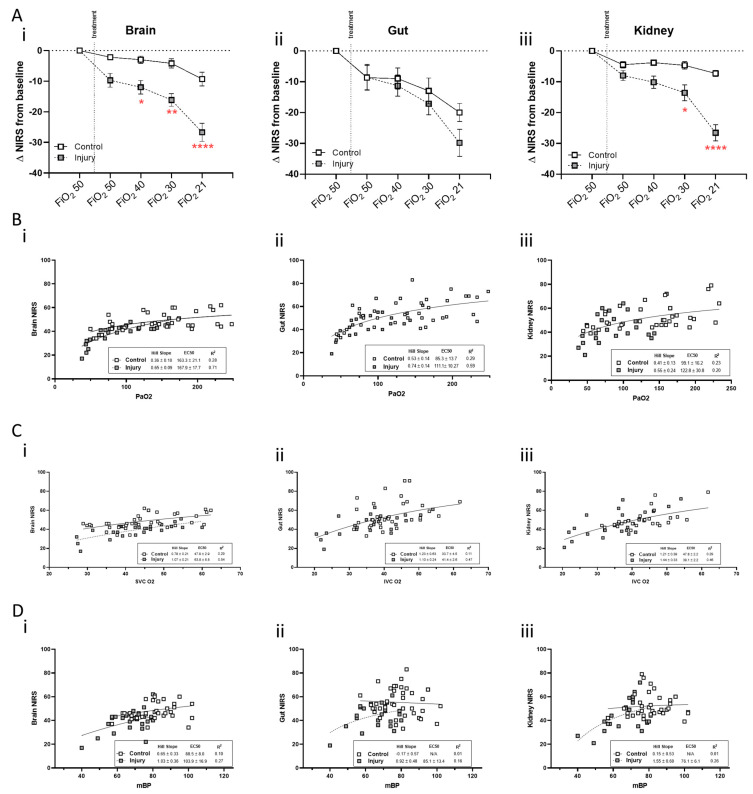
NIRS correlation to regional saturation. With each animal serving as its own control, the change from baseline rcSO_2_ (**Ai**), rgSO_2_ (**Aii**) and rrSO_2_ (**Aiii**) is plotted for 1 h post lung injury or sham and during FiO_2_ stepdown from 50 to 21%. (N = 6–7, * *p* < 0.05, ** *p* < 0.01, **** *p* < 0.0001). The correlations between PaO_2_ and regional NIRS are presented in (**Bi**–**Biii**). The correlation between regional PvO_2_ (SVC sample for brain NIRS; IVC sample for gut and kidney NIRS) and regional NIRS is shown in (**Ci**–**Ciii**). The correlation between mBP and regional NIRS is displayed in (**Di**–**Diii**).

Raw NIRS values for each site and measurement period are presented in Table 2. Significant interindividual variability is evident in regional saturations from all three regions, at every measurement period. The range of baseline (cerebral) rcSO_2_ was 44–62%, (gut) rgSO_2_ 41–90% and 46–79% (renal) rrSO_2_. Although between-group differences were only significant for rcSO_2_ at an FiO_2_ = 21%, regional saturations decreased modestly with decreasing FiO_2_ in each region under control conditions and more significantly in the OA group. Focusing on rcSO_2_ alone, NIRS readings in the control group remained within 10% of baseline when FiO_2_ was titrated between 50 and 30% and decreased by 18% when FiO_2_ = 21%. With OA injury, rcSO_2_ decreased by 20–48% from baseline, with FiO_2_ step decreases from 40 to 21%. Given the degree of baseline variability present, further analysis was undertaken with each animal serving as its own control. The change in regional saturations from baseline with step decreases in FiO_2_ were then analyzed, as seen in Figure 4.

At the 1 h post OA or sham injury time period, a small but insignificant decrease from baseline NIRS values was evident in all regions (Figure 4). With each step decrease in FiO_2_, the OA-injured group showed a greater decrease in rcSO_2_ compared to the control group (Figure 4(Ai)). Mesenteric saturation (rgSO_2_) decreased in both control and OA-injured animals to a similar degree following step decreases in FiO_2_ (Figure 4(Aii)). Renal regional saturation (rrSO_2_) changes in the OA-injured group mirrored those seen in the brain, with control rrSO_2_ unchanged from baseline (Figure 4(Aiii)). Regional cerebral saturations (rcSO_2_) correlated with both PaO_2_ (Figure 4(Bi–Biii)) and respective venous PvO_2_ (Figure 4(Ci–Ciii)). Regional cerebral saturations demonstrated a more significant correlation with mBP compared to either renal or mesenteric regions (Figure 4(Di–Diii)).

**Figure 5:**
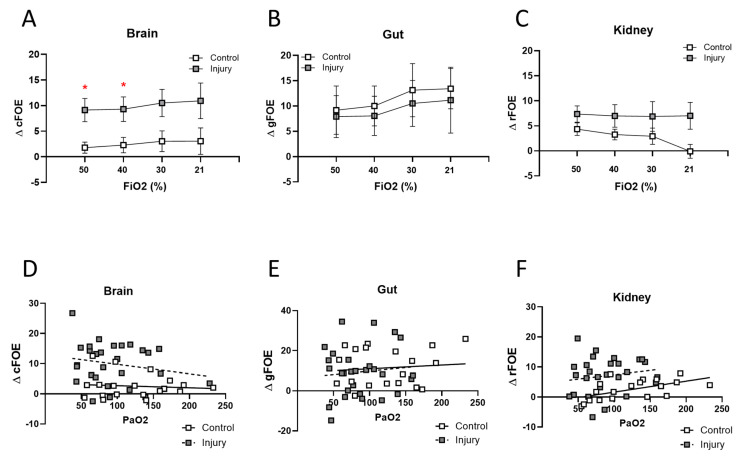
Fractional oxygen extraction. The change in fractional oxygen extraction (FOE) from baseline for each region: brain (**A**), gut (**B**) and kidney (**C**) is plotted 1 h post lung injury or sham and during FiO_2_ stepdown from 50 to 21%. For all measurement points obtained at all FiO_2_s, the change from baseline fractional oxygen extraction is plotted against PaO_2_ for brain (**D**), gut (**E**) and kidney (**F**) with slopes for lung injury or sham treatment group determined by simple linear regression (N = 6–7, * *p* < 0.05). Regression lines shown as solid line for control, and dotted line for ALI animals.

The change in fractional oxygen extraction (FOE) is presented in Figure 5. After OA injury, the change in cerebral FOE was significantly greater than that of the control group, while the change in FOE of either the renal or mesenteric regions did not differ between groups. Change in FOE was also analyzed by systemic PaO_2_; oxygen extraction in the cerebral circuit trended toward increasing at lower PaO_2_ values in the lung injury group compared to controls but not in renal or gut circulations where the slope was identical between treatment groups. NIRS-derived calculation of cerebral FOE was compared to blood gas-derived FOE at serial FiO_2_s, confirming that while the absolute ranges differ, these values have identical slope and thus are similarly affected by FiO_2_ (supplementary Figure S1).

In an attempt to determine an SaO_2_ threshold below which cerebral oxygenation becomes impaired, rcSO_2_ data were analyzed by the arterial saturation ranges 95–100%, 90–95%, 85–90%, 80–85% and <85% obtained after OA or sham injury in Table 3. One rcSO_2_ measurement was obtained at each of the four distinct arterial saturation ranges, resulting in n = 24 values from six control animals and n = 28 from seven OA animals in the left-hand panel. We chose an rcSO_2_ < 45% and a decrease in rcSO_2_ of >20% from baseline to denote potential risk. As expected, the number of animals in the control group who developed an arterial saturation below 90% was limited, although an SaO_2_ < 90% was observed in four control animals when FiO_2_ = 21%. Above an SaO_2_ of 90%, average rcSO_2_ was maintained at values > 45% in the control group, with a single measurement calculated to be 20% below baseline. In contrast, rcSO_2_ in the OA group was <45% at all ranges of SaO_2_ after injury. We then determined the number (%) of rcSO_2_ values in each saturation range that represented a ≥20% decrease from baseline in the right-hand panel. In the OA-injured groups, with data points that met the SaO_2_ threshold of ≥90%, 11/16 measurements (69%) were consistent with values > 20% below the individual baseline. In both control and OA-injured animals with an SaO_2_ threshold < 90%, the average rcSO_2_ was <45% and nearly all resulted in a 20% decrease from baseline.

**Figure 6:**
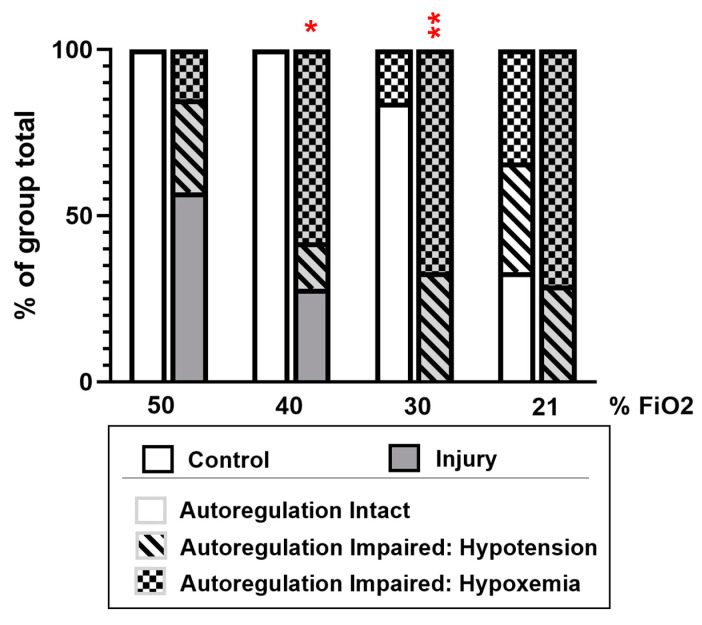
Cerebral autoregulation analysis. An autoregulation calculator was used to determine if cerebral autoregulation was intact or impaired due to either a drop in blood pressure or hypoxemia following stabilization for 1 h post lung injury or sham at an FiO_2_ of 50, 40, 30 and 21%. Comparisons were made between control and ALI animals by Chi-squared test. (N = 6–7, * *p* < 0.05, ** *p* < 0.01).

Using the autoregulation calculation algorithm designed for this study to correlate changes in rcSO_2_ with changes in blood pressure, PaCO_2_ and SaO_2_, setting a cut limit for significant change at 20% change from the trendline to detect deviations from established nomograms, we report that autoregulation was impaired after OA-induced lung injury as a function of blood pressure (a proxy for systemic blood flow) at all FiO_2_ steps; however, below an FiO_2_ of 40%, impaired autoregulation was predominantly due to hypoxemia (Figure 6).

## 4. Discussion

In a piglet model of moderate OA-induced ALI, we have demonstrated that cerebral blood flow regulation is dependent on both arterial blood pressure and arterial oxygenation, with oxygenation assuming a more predominant role when FiO_2_ decreases below 40%. When FiO_2_ is decreased from 50 to 21% under control conditions, a redistribution of blood from the mesentery to the cerebral and renal circulations was observed but was absent with OA ALI. In the OA-injured group, the average rcSO_2_ decreased to below 45% from the first measurement period at 1 h with FiO_2_ maintained at 50%. For the majority of measurements, this represented a decrease from baseline rcSO_2_ of more than 20%. Cerebral fractional oxygen extraction increased to a significantly greater extent than either kidney or mesentery at any delivered FiO_2_. As such, in this lung injury model, we were unable to define a specific systemic oxygen saturation threshold below which cerebral oxygen extraction increased or above which normal cerebral oxygenation was ensured. Given the variability in cerebral response to decreasing oxygen delivery, these findings suggest the need for an individualized approach to oxygen titration in neonatal ALI patients. The combination of NIRS and continuous blood pressure monitoring with TNE may be helpful in this regard [17].

### 4.1. Oleic Acid Lung Injury Model

The oleic acid model has been well documented to reproduce the pathophysiological characteristics of ALI: early and rapidly reversible patchy inflammatory lung injury with permeability changes and impairment in gas exchange and lung mechanics [18]. We used an oleic acid dose expected to induce a moderate degree of ALI to permit exploration of decreasing levels of oxygenation with limited hemodynamic compromise during the experiment. We based the OA dosing on our previously established porcine neonatal model of graded lung injury, where lung US scores were validated against standard histological assessment using the American Thoracic Society Acute Lung Injury in Animals consensus method [9]. The documented alterations in lung mechanics, P/F ratio, shunt fraction, arterial oxygenation and lung US scores are consistent with our previous moderate lung injury model, although the TNE changes observed in the present study are less impressive. OA infusion induces both local and systemic inflammatory effects manifested by increases in lung and serum inflammatory mediators, with hemodynamic consequences [19]. With more severe injury, systemic effects are evident: systemic vascular resistance increases, both RV and LV function and output are impaired, and arterial blood pressure falls. An increase in pulmonary artery pressure and pulmonary vascular resistance has been well documented in larger porcine models when pulmonary artery catheterization is possible [20]. In the neonatal piglet model after stable establishment of moderate or severe ALI, we previously reported decreased TAPSE as well as diminished RVO, without change in PAAT/ET [9]. TAPSE is described as a predictive marker of right ventricular dysfunction and hypotension [21,22]. In the present model, targeted neonatal echocardiography was performed at 1 h post injury with FiO_2_ = 50%. In the OA-injured group at this early time period, SVR, LV and RV outputs were unaltered, but the significant decrease in tricuspid annular plane systolic excursion suggests the onset of right ventricular strain consistent with the increased pulmonary vascular pressure and pulmonary vascular resistance observed in a more mature model. The possibility of direct cerebral effects of OA administration should also be considered, as a small number of studies document evidence of increased cerebral cytokine production, nitro-oxidative stress and cerebral edema in mature OA animal models [23]. Direct cerebral effects in OA injury models may potentially contribute to the significant cerebral desaturation discussed below but would require measurement of cerebral cytokines or histological assessment for confirmation.

### 4.2. NIRS Data

Normal neonatal regional cerebral saturations (rcSO_2_) are reported between 60 and 80%, with fractional oxygen extraction (FOE) of 20–40% [8]. In a piglet model, Hou et al. documented the occurrence of cerebral anaerobic metabolism with rSO_2_ < 45% and hypoxic ischemic encephalopathy with prolonged rcSO_2_ < 45%, consistent with neurological outcome studies in infants [24]. Kurth et al. found a lower cerebral hypoxia-ischemia rcSO_2_ threshold of 33–44% for brain metabolic dysfunction and time-dependent overt neurological injury in a similar piglet model [25]. Given the lack of consensus regarding a clear lower rcSO_2_ limit denoting brain at risk, and the significant inter-individual variability rcSO_2_ values that have been documented, Weber et al. suggest that a 20% decrease from baseline rcSO_2_ may be a more important indicator of a brain at potential risk of ischemic injury [8]. The average rcSO_2_ at baseline observed in our model (50.4 ± 6.7% and 54 ± 6.8% in the control and OA group, respectively) is lower than that reported by either Hou et al. [24] or in human neonates [8] but is similar to baseline values obtained in other piglet models [25,26,27]. Regional saturations correlated well with respective venous PvO_2_, validating the measurements obtained [28]. The algorithms used by different NIRS monitors, species differences and varying effects of differing anesthetic regimes on cerebral blood flow and metabolism may contribute to the variations in baseline rcSO_2_ reported between studies. While comparison to an awake rcSO_2_ would be considered the gold standard, animal movement precluded any ability to obtain reliable awake readings.

Using either an anaerobic threshold rcSO_2_ of 45% as suggested by Hou et al. [24], or a >20% decrease from baseline [8], to indicate onset of concern, our lung injury group could be considered to be at risk at any FiO_2_ between 50 and 21% (Table 2), even with arterial saturations maintained above 95% (Table 3). Control animals demonstrated rcSO_2_ values of concern at FiO_2_ = 21% when SaO_2_ fell below 90%. These results suggest the need to ensure stability of both hemodynamics and regional saturations when titrating FiO_2_ to lower SaO_2_ ranges in ALI patients, as cerebral oxygenation may be compromised with even modest decreases in arterial oxygenation. Evidence of increases in cerebral venous lactate or histological studies would be required to validate these findings; Heuer et al. [29] have also questioned whether the current recommendation to accept arterial saturations of >90% is adequate in ARDS patients, particularly in those with concomitant cerebral injury.

Evaluation of concurrent renal, mesenteric and cerebral regional saturations has been less extensively reported under hypoxic conditions. McNeill et al. [30] report baseline renal and mesenteric saturations between 64 and 87% and 32 and 66%, respectively, in line with the results obtained in the current study. Variability in the saturation ranges of the renal and mesenteric bed is reportedly greater than that of the cerebral territory [30] and is corroborated in the present work. Using multi-site NIRS monitoring, Montaldo et al. [31] demonstrated that during the post-delivery transition period in full term infants, rcSO_2_ increases more rapidly than rrSO_2_ and rgSO_2_, suggesting blood flow redistribution from the latter two beds such that oxygen delivery to the brain is preserved at the expense of kidneys and splanchnic tissue. Holler et al. [32] support a combination of peripheral and cerebral NIRS monitoring to determine redistribution of blood flow during ischemic or hypoxic periods. In the present study, centralization of the mesenteric circulation was only evident in the control group (Figure 4). With decreasing FiO_2_, mesenteric saturations decreased by more than 20%, while cerebral and renal sats showed minimal/modest change from baseline. Differential responses were not evident in the OA-injured group, as all regional saturations fell to a similar extent with decreasing FiO_2_, reflecting global hypoxic delivery to all vascular beds. Under these circumstances, fractional extraction of oxygen increased to a greater degree in the cerebral region, reflecting compensation for exhausted autoregulation.

### 4.3. Autoregulation

Cerebral autoregulation in neonates classically follows a sigmoidal curve, where cerebral blood flow does not vary with blood pressure along a defined range, but above and below which cerebral blood flow is pressure passive. Cerebral blood flow and oxygen delivery increase with hypoxia, and the pressure threshold above which autoregulation holds generally varies with oxygen saturation [33]. The flow augmentation effect of hypoxia opposes the flow dampening effect of hypocapnia, but effects of oxygen and carbon dioxide on autoregulation thresholds may be synergistic. In conscious humans it has been demonstrated that the control of cerebral blood flow by hypoxia exceeds its regulation by hypocapnia at the extremes of hypoxia, while increasing CO_2_ tensions augment the sensitivity of the cerebral blood flow response to hypoxia [34]. Cerebral flow pressure passivity can fluctuate in sick neonates, resulting in increased transient episodes of autoregulation loss without changes in oxygen or CO_2_ parameters [35].

Estimation of cerebral autoregulation by NIRS utilizes the absorption spectra of oxyhemoglobin to act as an endogenous tracer for arterial flow; adjusting for cerebral oxygen extraction, cerebral blood flow is calculated by a modified Fick method [36]. Calculation from cerebral oximetry metrics permits detection of impaired cerebral autoregulation in neonates and its causal attribution [37,38]. This NIRS method is also effective to discern regional blood flow and cerebral saturation in neonatal piglets, despite impedance from their thicker calvarium [27]. To further analyze these data, we used an autoregulation calculation algorithm designed to best fit changes in cerebral SO_2_ with contemporaneous changes in blood pressure, PaCO_2_ and/or PO_2_ across a range of FiO_2_ values, using established nomograms from large samples of neonatal populations [14,15,16]. The output (Figure 6) identifies the proportion of dynamic autoregulation failure attributable primarily to pressure variation, O_2_ tension variation or to CO_2_ variation, by curve fitting to these nomograms [13]. As a computational model, the algorithm must still be externally validated, but these results track with the manually calculated comparisons made in Figure 4 and Figure 5 and serve to integrate these findings. The specific attribution of autoregulation impairment in this analysis remains speculative and should trigger further study.

Despite observing increased fractional oxygen extraction in the ALI brain, we found onset of pressure passive cerebral flow at higher FiO_2_ and at higher systemic SaO_2_ in the ALI group compared to control. Loss of autoregulation attributable to hypotension was a greater feature of ALI circulation than of control circulation at all oxygen tensions in individual cases. In infants undergoing anesthesia, intraoperative hypotension >20% below baseline may be associated with decreased cerebral blood flow [39]. This finding has been investigated in neonatal piglet models, showing decreased cerebral tissue oxygenation following hypovolemic hypotension [40], but with the loss of cerebral autoregulation specifically attributed to pressure fluctuation rather than loss of oxygen carrying capacity [41]. While hypocapnia is known to augment the impact of hypotension on cerebral blood flow and thus complicate assessment of autoregulation [42], none of our animals were hypocapnic; the ALI group had a small but statistically significant increase in PCO_2_ at all FiO_2_ levels. Thus, none of the loss of cerebral autoregulation we observed could be attributed to CO_2_ fluctuation. A large analysis of human infant data indicated that mild hypotension triggers decreased cerebral metabolic rate, resulting in maintenance of cerebral tissue oxygenation despite diminished perfusion pressure; in severe hypotension, cerebral metabolic reserve is limited, and loss of autoregulation is pathological [43]. In our ALI model, capillary leak does result in a degree of hypovolemic hypotension, albeit with preserved hemoglobin. Systemic blood flow fluctuations may contribute towards pressure passivity of cerebral blood flow at higher oxygen saturation than in controls. Our data in sum are consistent with the finding that hemodynamic instability in patients with acute lung injury impairs cerebral homeostatic mechanisms, resulting in a vulnerability to brain injury independent of the effects of inflammation or hypoxemia [44].

### 4.4. Limitations

Our sample size was small with significant baseline variability. However, using a baseline rcSO_2_ of 50 ± 5% and assuming a modest reduction in rcSO_2_ of 15% with OA injury, α = 0.05 and power = 0.8, we calculated that a sample size of 12 subjects would be required. The actual reduction in baseline rcSO_2_ was 20%, suggesting an adequate number of animals was studied, in alignment with ethical principles for animal research.

No animal model reproduces all the characteristics of ALI/ARDS in humans. Oleic acid is a fatty acid that causes direct toxicity to the pulmonary endothelium. The pathophysiology of the OA injury might differ from the more common causes of ALI such as aspiration or sepsis in the term neonate, but the pulmonary pathology is similar. Measurement of serum lactate and/or direct measurement of tissue oxygenation would be required to confirm when the significant regional desaturations observed with OA injury were associated with a switch to anaerobic metabolism. As such, caution with translation of these findings to the clinical setting is recommended without corroboration in additional pre-clinical models and further study in the neonatal ICU setting.

## 5. Conclusions

We conclude that in an animal model of neonatal ALI, cerebrovascular blood flow regulation is dependent on both arterial blood pressure and oxygenation but primarily dependent on oxygenation when FiO_2_ < 40%. We could not define an oxygenation threshold below which cerebral oxygen extraction increases or above which baseline cerebral oxygenation is maintained. Rather, cerebral oxygen extraction is enhanced from the onset of injury, reflecting compensation for exhausted cerebral autoregulation as a mechanism to protect against tissue hypoxia. Continuous multi-site NIRS monitoring combined with arterial blood pressure measurements and TNE assessment of cardiac filling/function may be helpful when managing patients with ALI to follow trends in tissue oxygenation when titrating FiO_2_. The lower saturation ranges recommended for premature neonatal care may not be applicable to term infants with acute lung injury.

## Figures and Tables

**Table 1:** Group characteristics.

		Control					Injury				
		Baseline	1 h Post	40% FiO_2_	30% FiO_2_	21% FiO_2_	Baseline	1 h Post	40% FiO_2_	30% FiO_2_	21% FiO_2_
**weight**	mean	2.13					2.177				
	SE	0.09					0.146				
**HR**	mean	155.7	155.5	157.8	154.8	171.2	157.9	154.4	162.0	161.0	187.6
	SE	14.3	14.3	17.1	21.0	25.7	5.0	10.7	10.7	15.7	18.0
**BP**	mean	84.3	81.7	78.8	74.8	74.0	77.3	70.7	68.4	71.6	70.3
	SE	4.3	4.8	5.4	5.5	7.1	3.3	3.0	3.3	5.6	7.1
**Systolic BP**	mean	113.2	109.7	106.5	101.6	101.0	102.3	94.9 *	94.4	92.9	93.6
	SE	5.6	5.1	5.1	5.2	7.8	4.2	3.2	4.9	5.3	7.2
**Diastolic BP**	mean	65.3	64.7	62.2	59.6	58.4	59.9	54.1	55.6	56.9	54.7
	SE	3.1	5.6	6.2	6.7	7.5	2.6	2.8	5.5	5.7	7.0
**CVP**	mean	7.7	7.5	7.3	7.2	7.2	6.4	6.1	5.9	5.9	5.9
	SE	0.6	0.3	0.4	0.5	0.6	0.5	0.4	0.4	0.4	0.4
**temp**	mean	36.7	37.9	37.5	37.4	37.1	36.7	37.7	37.7	37.6	37.5
	SE	0.2	0.3	0.2	0.2	0.2	0.2	0.3	0.2	0.2	0.2
**PI**	mean	0.72	0.80	0.80	0.79	0.81	0.90	0.56	0.72	0.56	0.57
	SE	0.11	0.14	0.13	0.15	0.15	0.14	0.07	0.09	0.06	0.08

Weight (kg), heart rate (HR; beats per minute), mean, systolic and diastolic blood pressure (BP; mmHg), central venous pressure (CVP; mmHg), temperature (°C) and perfusion index (PI) were documented at baseline, 1 h post lung injury or sham and post stabilization following stepwise decrease in FiO_2_ from 50%, to 40%, to 30% and then 21% (presented as mean ± SE, N = 6–7, * *p* < 0.05).

**Table 2:** Regional NIRS.

	rcSO_2_		rgSO_2_		rrSO_2_	
FiO_2_	Control	OA	Control	OA	Control	OA
**Baseline 50%**	50.4 (2.7)	54.6 (2.6)	66.5 (6.2)	61.9 (4.4)	47.7 (6.0)	60.6 (5.0)
**1 h 50%**	48.3 (2.6)	44.9 (1.4)	57.8 (7.4)	53.3 (2.7)	48.6 (2.7)	52.6 (5.7)
**40%**	47.5 (2.5)	42.6 (0.7)	57.7 (5.8)	50.6 (2.0)	49.6 (2.4)	50.4 (4.2)
**30%**	46.3 (2.6)	38.4 (1.0)	53.5 (4.1)	44.7 (2.3)	48.6 (2.5)	47.0 (4.0)
**21%**	41.2 (3.2)	27.9 (2.6) **	46.5 (4.7)	32.0 (2.5)	45.4 (2.8)	34.0 (3.2)

Average (SE) NIRS values for each region at baseline, 1 h post OA lung or sham injury and at each step decrease in FiO_2_ are displayed. (N = 6–7, ** *p* < 0.01).

**Table 3:** Regional NIRS data grouped by SaO_2_ range.

SaO_2_ Range (Post Injury or Sham)	N		rcSO_2_ Post Injury		rcSO_2_ > 20% Decrease	
	C	OA	C	OA	C	OA
**95–100%**	17	11	48.5 (1.6)	44.1(1.0)	1 ( 5%)	7 (64%)
**90–95%**	3	5	47.3 (3.5)	40.8 (0.8)	0 (0%)	4 (80%)
**85–90%**	1	3	37 (0)	35.5 (1.2)	1 (100%)	3 (100%)
**<85%**	3	9	36.6 (2.7)	30.1 (2.6)	2 (67%)	8 (89%)

Middle panel shows average (SE) rcSaO_2_ in control and OA-injured animals obtained after OA or sham injury at each SaO_2_ range. Right hand panel shows the number and % of NIRS readings that represent a 20% decrease from baseline in each group (N = 6–7; n = 24–28).

## Data Availability

The original contributions presented in the study are included in the article/Supplementary Materials, further inquiries can be directed to the corresponding author.

## Supplementary Materials

The following supporting information can be downloaded at: https://www.mdpi.com/article/10.3390/children11050611/s1, Figure S1: Calculated Change in Fractional Oxygen Extraction.

## References

[1] De Luca D., van Kaam A.H., Tingay D.G., Courtney S.E., Danhaive O., Carnielli V.P., Zimmermann L.J., Kneyber M.C.J., Tissieres P., Brierley J. (2017). The Montreux definition of neonatal ARDS: Biological and clinical background behind the description of a new entity. Lancet Respir. Med..

[2] Verheijen A.C., Janssen E.E.R., van der Putten M.E., van Horck M.W.P., van Well G.T.J., Van Loo I.H.M., Hutten M.C., Van Mechelen K. (2022). Management of severe neonatal respiratory distress due to vertical transmission of severe acute respiratory syndrome coronavirus 2: A case report. J. Med. Case Rep..

[3] Saugstad O.D. (2004). The role of oxygen in neonatal resuscitation. Clin. Perinatol..

[4] Cummings J.J., Polin R.A. (2016). Oxygen Targeting in Extremely Low Birth Weight Infants. Pediatrics.

[5] Kayton A., Timoney P., Vargo L., Perez J.A. (2018). A Review of Oxygen Physiology and Appropriate Management of Oxygen Levels in Premature Neonates. Adv. Neonatal Care Off. J. Natl. Assoc. Neonatal Nurses.

[6] Sweet D.G., Carnielli V., Greisen G., Hallman M., Ozek E., Te Pas A., Plavka R., Roehr C.C., Saugstad O.D., Simeoni U. (2019). European Consensus Guidelines on the Management of Respiratory Distress Syndrome—2019 Update. Neonatology.

[7] Elsayed Y.N., Fraser D. (2016). Integrated Evaluation of Neonatal Hemodynamics Program Optimizing Organ Perfusion and Performance in Critically Ill Neonates, Part 1: Understanding Physiology of Neonatal Hemodynamics. Neonatal Netw. NN.

[8] Weber F., Scoones G.P. (2019). A practical approach to cerebral near-infrared spectroscopy (NIRS) directed hemodynamic management in noncardiac pediatric anesthesia. Paediatr. Anaesth..

[9] Elsayed Y.N., Hinton M., Graham R., Dakshinamurti S. (2020). Lung ultrasound predicts histological lung injury in a neonatal model of acute respiratory distress syndrome. Pediatr. Pulmonol..

[10] Moodley Y., Sturm M., Shaw K., Shimbori C., Tan D.B., Kolb M., Graham R. (2016). Human mesenchymal stem cells attenuate early damage in a ventilated pig model of acute lung injury. Stem Cell Res..

[11] Matute-Bello G., Downey G., Moore B.B., Groshong S.D., Matthay M.A., Slutsky A.S., Kuebler W.M. (2011). An official American Thoracic Society workshop report: Features and measurements of experimental acute lung injury in animals. Am. J. Respir. Cell Mol. Biol..

[12] Sidorenko I., Turova V., Botkin N., Eckardt L., Alves-Pinto A., Felderhoff-Muser U., Rieger-Fackeldey E., Kovtanyuk A., Lampe R. (2018). Modeling Cerebral Blood Flow Dependence on Carbon Dioxide and Mean Arterial Blood Pressure in the Immature Brain With Accounting for the Germinal Matrix. Front. Neurol..

[13] Elsayed Y.N., Dakshinamurti S. (2021). Titration of inspired oxygen in preterm infants with hypoxemic respiratory failure using near-infrared spectroscopy and pulse oximetry: A new approach. Pediatr. Pulmonol..

[14] Alderliesten T., Dix L., Baerts W., Caicedo A., van Huffel S., Naulaers G., Groenendaal F., van Bel F., Lemmers P. (2016). Reference values of regional cerebral oxygen saturation during the first 3 days of life in preterm neonates. Pediatr. Res..

[15] Zubrow A.B., Hulman S., Kushner H., Falkner B. (1995). Determinants of blood pressure in infants admitted to neonatal intensive care units: A prospective multicenter study. Philadelphia Neonatal Blood Pressure Study Group. J. Perinatol. Off. J. Calif. Perinat. Assoc..

[16] Altaany D., Natarajan G., Gupta D., Zidan M., Chawla S. (2015). Severe Intraventricular Hemorrhage in Extremely Premature Infants: Are high Carbon Dioxide Pressure or Fluctuations the Culprit?. Am. J. Perinatol..

[17] Amer R., Kalash R., Seshia M.M., Elsayed Y.N. (2017). The Impact of Integrated Evaluation of Hemodynamics on Management of Preterm Infants with Late-Onset Compromised Systemic Circulation. Am. J. Perinatol..

[18] Matute-Bello G., Frevert C.W., Martin T.R. (2008). Animal models of acute lung injury. Am. J. Physiol. Lung Cell. Mol. Physiol..

[19] Tisoncik J.R., Korth M.J., Simmons C.P., Farrar J., Martin T.R., Katze M.G. (2012). Into the eye of the cytokine storm. Microbiol. Mol. Biol. Rev. MMBR.

[20] Graham M.R., Gulati H., Kha L., Girling L.G., Goertzen A., Mutch W.A. (2011). Resolution of pulmonary edema with variable mechanical ventilation in a porcine model of acute lung injury. Can. J. Anaesth.=J. Can. D’Anesth..

[21] Gulasti F., Gulasti S., Sari S. (2023). Tricuspid annular plane systolic excursion to predict arterial hypotension caused by general anesthesia induction. Minerva Anestesiol..

[22] Giovanardi P., Tincani E., Maioli M., Tondi S. (2020). The Prognostic Importance of TAPSE in Early and in Stable Cardiovascular Diseases. J. Cardiovasc. Dev. Dis..

[23] Kamuf J., Garcia-Bardon A., Ziebart A., Thomas R., Folkert K., Frauenknecht K., Thal S.C., Hartmann E.K. (2018). Lung injury does not aggravate mechanical ventilation-induced early cerebral inflammation or apoptosis in an animal model. PLoS ONE.

[24] Hou X., Ding H., Teng Y., Zhou C., Tang X., Li S. (2007). Research on the relationship between brain anoxia at different regional oxygen saturations and brain damage using near-infrared spectroscopy. Physiol. Meas..

[25] Kurth C.D., McCann J.C., Wu J., Miles L., Loepke A.W. (2009). Cerebral oxygen saturation-time threshold for hypoxic-ischemic injury in piglets. Anesth. Analg..

[26] Ringer S.K., Clausen N.G., Spielmann N., Weiss M. (2019). Effects of moderate and severe hypocapnia on intracerebral perfusion and brain tissue oxygenation in piglets. Paediatr. Anaesth..

[27] Silvera F., Gagliardi T., Vollono P., Fernandez C., Garcia-Bayce A., Berardi A., Badia M., Beltran B., Cabral T., Abella P. (2022). Study of the relationship between regional cerebral saturation and pCO2 changes during mechanical ventilation to evaluate modifications in cerebral perfusion in a newborn piglet model. Braz. J. Med. Biol. Res.=Rev. Bras. Pesqui. Medicas Biol..

[28] Chen M.W., Reyes M., Kulikowicz E., Martin L., Hackam D.J., Koehler R.C., Lee J.K. (2018). Abdominal near-infrared spectroscopy in a piglet model of gastrointestinal hypoxia produced by graded hypoxia or superior mesenteric artery ligation. Pediatr. Res..

[29] Heuer J.F., Pelosi P., Hermann P., Perske C., Crozier T.A., Bruck W., Quintel M. (2011). Acute effects of intracranial hypertension and ARDS on pulmonary and neuronal damage: A randomized experimental study in pigs. Intensive Care Med..

[30] McNeill S., Gatenby J.C., McElroy S., Engelhardt B. (2011). Normal cerebral, renal and abdominal regional oxygen saturations using near-infrared spectroscopy in preterm infants. J. Perinatol. Off. J. Calif. Perinat. Assoc..

[31] Montaldo P., De Leonibus C., Giordano L., De Vivo M., Giliberti P. (2015). Cerebral, renal and mesenteric regional oxygen saturation of term infants during transition. J. Pediatr. Surg..

[32] Holler N., Urlesberger B., Mileder L., Baik N., Schwaberger B., Pichler G. (2015). Peripheral Muscle Near-Infrared Spectroscopy in Neonates: Ready for Clinical Use? A Systematic Qualitative Review of the Literature. Neonatology.

[33] Rhee C.J., da Costa C.S., Austin T., Brady K.M., Czosnyka M., Lee J.K. (2018). Neonatal cerebrovascular autoregulation. Pediatr. Res..

[34] Mardimae A., Balaban D.Y., Machina M.A., Battisti-Charbonney A., Han J.S., Katznelson R., Minkovich L.L., Fedorko L., Murphy P.M., Wasowicz M. (2012). The interaction of carbon dioxide and hypoxia in the control of cerebral blood flow. Pflug. Arch. Eur. J. Physiol..

[35] Soul J.S., Hammer P.E., Tsuji M., Saul J.P., Bassan H., Limperopoulos C., Disalvo D.N., Moore M., Akins P., Ringer S. (2007). Fluctuating pressure-passivity is common in the cerebral circulation of sick premature infants. Pediatr. Res..

[36] Pryds O., Edwards A.D. (1996). Cerebral blood flow in the newborn infant. Arch. Dis. Child. Fetal Neonatal Ed..

[37] Pezzato S., Govindan R.B., Bagnasco F., Panagopoulos E.M., Robba C., Beqiri E., Smielewski P., Munoz R.A., d’Udekem Y., Moscatelli A. (2023). Cerebral autoregulation monitoring using the cerebral oximetry index after neonatal cardiac surgery: A single-center retrospective cohort study. J. Thorac. Cardiovasc. Surg..

[38] Kooi E.M.W., Verhagen E.A., Elting J.W.J., Czosnyka M., Austin T., Wong F.Y., Aries M.J.H. (2017). Measuring cerebrovascular autoregulation in preterm infants using near-infrared spectroscopy: An overview of the literature. Expert Rev. Neurother..

[39] Michelet D., Arslan O., Hilly J., Mangalsuren N., Brasher C., Grace R., Bonnard A., Malbezin S., Nivoche Y., Dahmani S. (2015). Intraoperative changes in blood pressure associated with cerebral desaturation in infants. Paediatr. Anaesth..

[40] Ringer S.K., Clausen N.G., Spielmann N., Ohlerth S., Schwarz A., Weiss M. (2018). Effects of moderate and severe arterial hypotension on intracerebral perfusion and brain tissue oxygenation in piglets. Br. J. Anaesth..

[41] Hahn G.H., Heiring C., Pryds O., Greisen G. (2012). Cerebral vascular effects of hypovolemia and dopamine infusions: A study in newborn piglets. Acta Paediatr..

[42] Ringer S.K., Ohlerth S., Carrera I., Mauch J., Spielmann N., Bettschart-Wolfensberger R., Weiss M. (2016). Effects of hypotension and/or hypocapnia during sevoflurane anesthesia on perfusion and metabolites in the developing brain of piglets-a blinded randomized study. Paediatr. Anaesth..

[43] Rhondali O., Andre C., Pouyau A., Mahr A., Juhel S., De Queiroz M., Rhzioual-Berrada K., Mathews S., Chassard D. (2015). Sevoflurane anesthesia and brain perfusion. Paediatr. Anaesth..

[44] Ziaka M., Exadaktylos A. (2022). ARDS associated acute brain injury: From the lung to the brain. Eur. J. Med. Res..

