# Supplementary figures and images for: Assessment of Autoregulation of the Cerebral Circulation during Acute Lung Injury in a Neonatal Porcine Model
Source: Children (Basel). 2024 May 20;11(5):611. doi: 10.3390/children11050611 (PMC11119854; doi:10.3390/children11050611)

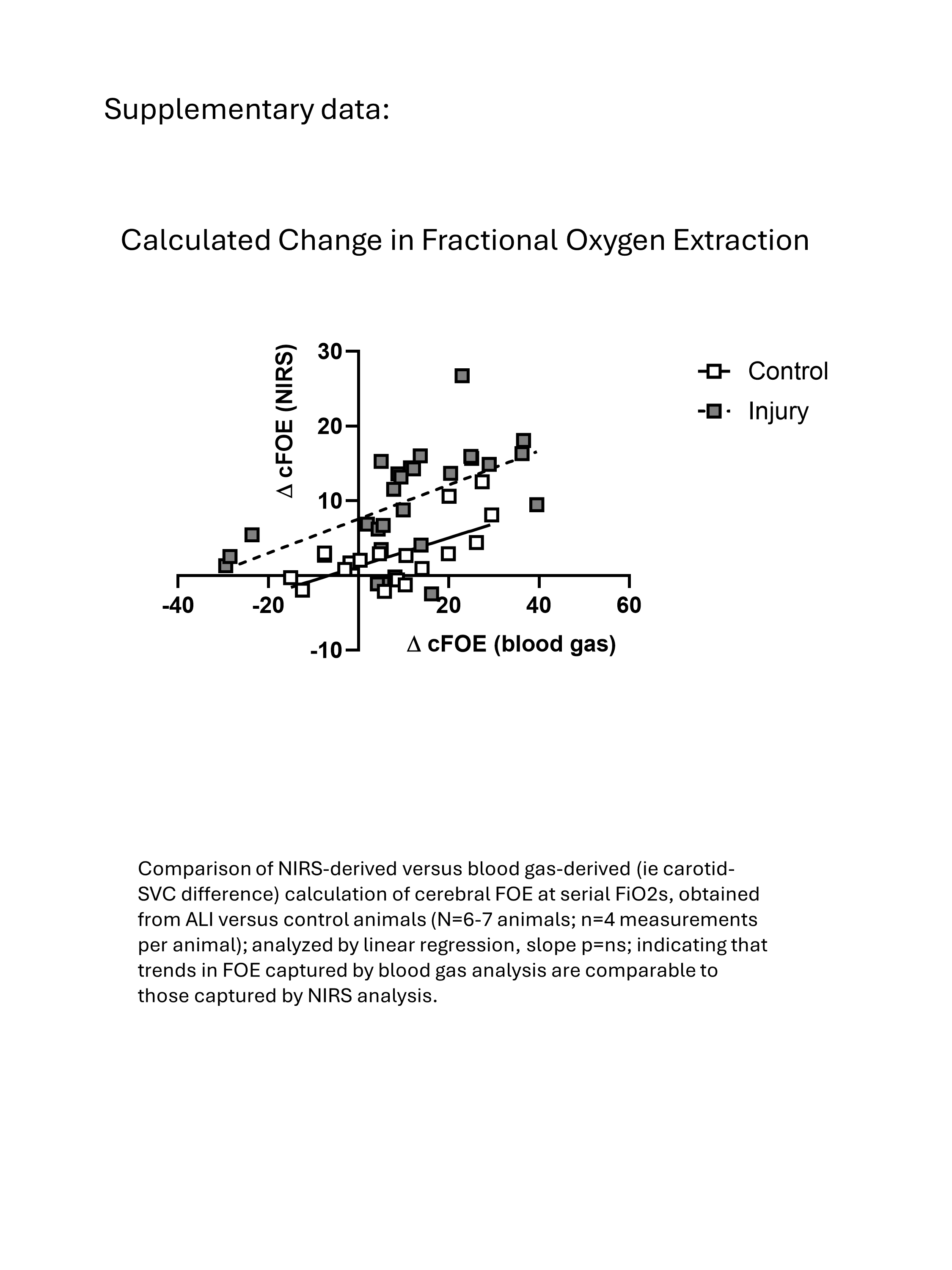

Supplement: Supplementary file 1 [file children-11-00611-s001.zip › children-2972675-supplementary.tif]
